# Continuous cuffless blood pressure monitoring using photoplethysmography-based PPG2BP-net for high intrasubject blood pressure variations

**DOI:** 10.1038/s41598-023-35492-y

**Published:** 2023-05-27

**Authors:** Jingon Joung, Chul-Woo Jung, Hyung-Chul Lee, Moon-Jung Chae, Hae-Sung Kim, Jonghun Park, Won-Yong Shin, Changhyun Kim, Minhyung Lee, Changwoo Choi

**Affiliations:** 1 Department of Electrical and Electronics Engineering, Chung-Ang University, Seoul, 06974 South Korea; 2 Department of Anesthesiology and Pain Medicine, Seoul National University Hospital, Seoul, 03080 South Korea; 3 Department of Industrial Engineering, Seoul National University, Seoul, 08826 South Korea; 4 School of Mathematics and Computing (Computational Science and Engineering), Yonsei University, Seoul, 03722 South Korea; 5 Pohang University of Science and Technology (POSTECH) (Artificial Intelligence), Pohang, 37673 South Korea; 6 Sky Labs Inc., Seongnam, 13486 South Korea

**Keywords:** Electrical and electronic engineering, Optoelectronic devices and components, Scientific data, Computational biophysics

## Abstract

Continuous, comfortable, convenient (C3), and accurate blood pressure (BP) measurement and monitoring are needed for early diagnosis of various cardiovascular diseases. To supplement the limited C3 BP measurement of existing cuff-based BP technologies, though they may achieve reliable accuracy, cuffless BP measurement technologies, such as pulse transit/arrival time, pulse wave analysis, and image processing, have been studied to obtain C3 BP measurement. One of the recent cuffless BP measurement technologies, innovative machine-learning and artificial intelligence-based technologies that can estimate BP by extracting BP-related features from photoplethysmography (PPG)-based waveforms have attracted interdisciplinary attention of the medical and computer scientists owing to their handiness and effectiveness for both C3 and accurate, i.e., C3A, BP measurement. However, C3A BP measurement remains still unattainable because the accuracy of the existing PPG-based BP methods was not sufficiently justified for *subject-independent* and *highly varying* BP, which is a typical case in practice. To circumvent this issue, a novel convolutional neural network(CNN)- and calibration-based model (PPG2BP-Net) was designed by using a comparative paired one-dimensional CNN structure to estimate highly varying intrasubject BP. To this end, approximately $70\%$, $20\%$, and $10\%$ of 4185 cleaned, independent subjects from 25,779 surgical cases were used for training, validating, and testing the proposed PPG2BP-Net, respectively and exclusively (i.e., subject-independent modelling). For quantifying the intrasubject BP variation from an initial calibration BP, a novel ‘standard deviation of subject-calibration centring (SDS)’ metric is proposed wherein high SDS represents high intrasubject BP variation from the calibration BP and vice versa. PPG2BP-Net achieved accurately estimated systolic and diastolic BP values despite high intrasubject variability. In 629-subject data acquired after 20 minutes following the A-line (arterial line) insertion, low error mean and standard deviation of $0.209\pm 7.509$ and $0.150\pm 4.549\;\textrm{mmHg}$ for highly varying A-line systolic and diastolic BP values, respectively, where their SDSs are 15.375 and 8.745. This study moves one step forward in developing the C3A cuffless BP estimation devices that enable the push and agile pull services.

## Introduction

Accurate and continuous self-monitoring of blood pressure (BP) is essential for healthy living, and cuff-based manometry has been widely employed to monitor cardiovascular status. However, cuff-based BP measurement could be inaccurate even in the clinic owing to sporadic phenomena (e.g., white-coat hypertension [hypertension only at office], masked hypertension [no hypertension at office]). The cuff-based BP measurements are widely used (e.g., office, home, and ambulatory BP measurements), but typically measure discontinuous BP accurately only when the BP is stable, and are sensitive to the cuff size and position. Patients desire continuous, comfortable, convenient (C3), and accurate (C3A) methods to measure and monitor their BP for early diagnosis of various cardiovascular diseases. Thus, a noninvasive technique to capture BP-related bio-waveforms seemed desirable, and motivated the rapid development of cuffless BP estimation and monitoring systems. Since 1896, enormous home healthcare applications have emerged from the ‘Riva Rocci mercury sphygmomanometer’ to the cuffless BP measurement systems, using ‘smart’ devices, such as a phone^1^, watch^2^, and wristlet,^3,4^; however, sufficient accuracy is not guaranteed^1,3^, and comfortable and convenient measurement is restricted owing to the 18 required sensors^4^. Thus, continuous and accurate self-measurement of existing BP (cuffless and noninvasive) remains a challenging task^2^.

From the seminal work^5^ in 2003, the potential capability of photoplethysmography (PPG)-based BP estimation has been increasingly revealed. As one of the pulse wave analysis methods, since a PPG signal can be readily obtained from a single light portable and wearable body sensor, e.g., a ring^6,7^, the PPG-based method is relevant for C3 BP measurement compared to the pulse transit/arrival time-based methods that typically require multiple electrocardiogram (ECG) and/or PPG sensors^8^. The PPG signals were successfully used to estimate systolic BP (SBP) of patients undergoing surgery (i.e., relaxed C3 conditions) along with the ECG signals based on the pulse arrival time^9,10^. Curve-fitting BP model parameters from PPG-extracted variables were initially used to estimate BP,^3^ and the feasibility was verified based on morphologic correlation between BP and PPG waveforms^11–13^.

Moreover, PPG waveforms have been successfully used to detect atrial fibrillation^6,7^. However, direct estimation of the BP from volatile PPG waveforms that are vulnerable to various interventions, such as physical exercise, posture, Valsalva manoeuvre, cold pressure, mental arithmetic, relaxation, amyl nitrate, anaesthesia, isometric exercise, and sustained handgrip^14^, remains a considerable task. Various non-parametric learning methods have been extensively applied to PPG-based BP estimation^15^. A plethora of innovative machine learning and artificial intelligence technologies can extract BP-related features from PPG waveforms^8,16^, e.g., deep belief network-restricted Boltzmann machines (DBN-RBM)^17^, artificial neural networks (ANN)^18^, support vector regression (SVR)^19–25^, decision tree regression (DTR)^23,24^, random forest regression (RFR)^24–26^, adaptive boosting regression (AdaboostR)^24^, convolutional neural network (CNN)^27^, CNN long short-term memory (CNN-LSTM)^28^, long-term recurrent convolutional network (LRCN)^29^, receptive field parallel attention shrinkage network (RFPASN)^30^, and concatenated CNN (Concat-CNN)^31^. The part^18,19,27–31^ of them fulfills the Association for the Advance of Medical Instrumentation (AAMI) standard successfully. However, since the previous methods^22,23,25,28–31^ were modeled and evaluated ‘subject-dependently,’ C3A BP measurement may not be guaranteed for highly variable inter-subject BP. Furthermore, the study^27^ was validated with relatively low intrasubject BP deviation, and some studies^17–20,22–26,28,31^ used insufficient training and validation subjects which may mislead the BP estimation, resulting in nonfulfillment of the AAMI^17,20,22,24,26^ (please refer to Table 3 for the details).

This study was conducted to evaluate a learning-based cuffless BP estimation system with calibration in challenging circumstances (i.e., highly varying intrasubject BP; Fig. 1). Here, we design a novel one-dimensional CNN (1D-CNN)-based network (‘PPG2BP-Net’) that can efficiently extract BP from PPG signals using a comparative paired 1D-CNN structure with calibration. Here, the calibration is required to improve the BP estimation accuracy^8^. To effectively train the designed PPG2BP-Net, the modelling data were preprocessed through: (i) abnormal surgical case elimination, (ii) downsampling and segmentation, (iii) abnormal segment elimination, (iv) normalization, and (v) balancing the number of segments. Throughout the preprocessing, 4185 clean subjects of the 4221 clean cases were obtained from 25, 779 surgical cases. From the 4185 clean subjects with A-line (arterial line) BP (ABP) waveforms, 2987 training ($\sim 70\%$) and 410 validation ($\sim 10\%$) subjects are randomly selected for the designed PPG2BP-Net to estimate the SBP and diastolic BP (DBP). From training and validation sets with a sufficient number of subjects, the PPG2BP-Net can overcome the limitation of possible misleading BP estimation. For the holdout validation, the PPG2BP-Net used the exclusively separated 797 test subjects ($\sim 20\%$) from the 4185 subjects. The comparative study with subject-independent modelling verified that the proposed PPG2BP-Net cuffless BP estimation system achieves considerably accurate SBP and DBP estimated values that completely fulfil the AAMI standard and attain Grade A British Hypertension Society (BHS) standard. For example, by testing 629 test subjects acquired after 20 minutes from the A-line insertion, the obtained mean error (ME) and standard deviation (SD) of estimated BP error are $0.209\pm 7.509$ and $0.150\pm 4.549\;\textrm{mmHg}$ for highly varying A-line SBP and DBP, respectively. From the observation that the conventional SD metric is relevant merely for calibration-free BP estimator’s performance evaluation, a novel ‘SD of subject-calibration centring (SDS)’ metric was proposed to quantify the intrasubject BP variation from an initially calibrated BP. Using the novel SDS metric, we can circumvent two potential practical issues in the design of calibration-based BP estimator: a *nonregenerative* issue, wherein a well-designed calibration-based BP estimator for high SD BP does not guarantee high performance for estimating high SDS BP with high intrasubject variability; and an *overqualified* issue, wherein the BP estimation performance is overqualified owing to the ambiguity of the conventional SD metric that does not clearly capture the intrasubject BP deviation.

In summary, the proposed PPG2BP-Net is modelled and evaluated with data from a sufficient sample (4185 subjects) with highly varying intrasubject BP and fulfils the AAMI and BHS standards. We surmise that the cuffless BP monitor based on the proposed PPG2BP-Net can provide a robust solution to measure varying BP accurately in new daily users as the proposed subject-independent approach is regenerative for a new subject. The cuffless BP measurement is tractable and enables 24-hour continuous measurement, BP variability assessment, and nocturnal BP monitoring during sleep. Therefore, the proposed PPG2BP-Net-based cuffless BP measurement has high potential to improve hypertension awareness, treatment, and management to enable early prediction of cardiovascular events. This study provides a prospect of the C3A cuffless BP estimation devices and their potential services.

## Results

**Figure 1:**
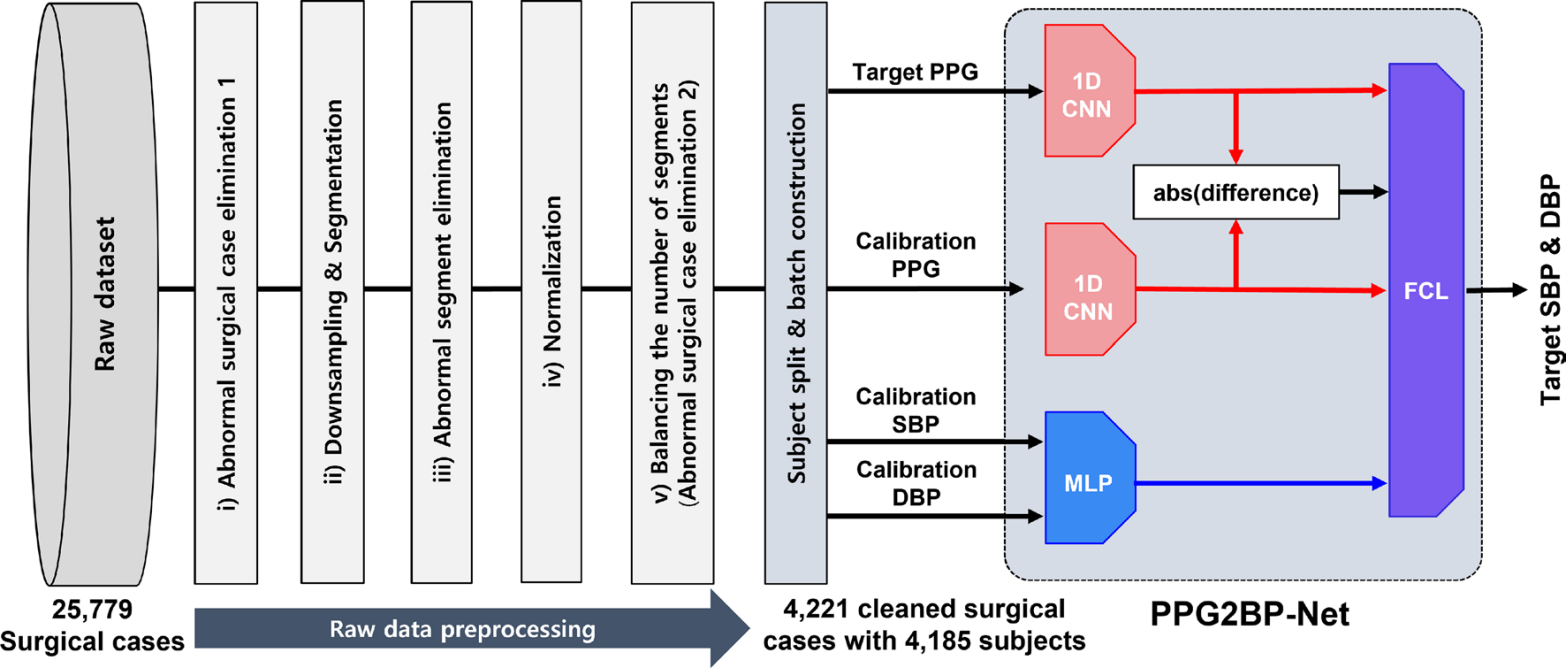
The proposed data preprocessing and PPG2BP-Net models for cuffless BP estimation.

### Characteristics of dataset

Raw, vital waveforms of 25, 779 surgical cases were acquired intraoperatively from Seoul National University Hospital (SNUH) between 2016 and 2019 for 4 years^32^ and included adult patients (age 18–90 years) for whom ABP was monitored intraoperatively. Among them, 4185 clean, independent subjects of the cleaned 4221 cases were enrolled (Fig. 1) through raw data preprocessing: (i) abnormal surgical case elimination, (ii) downsampling and segmentation, (iii) abnormal segment elimination, (iv) normalization, and (v) balancing the number of segments. These 4185 clean subjects were stratified as 2978, 410, and 797 subjects for training, validation, and test (approximately $70\%$, $10\%$, and $20\%$ of the 4185 subjects), respectively.

**Table 1:** Characteristics of the subject data that were analyzed in this study. ‘Whole set’ includes whole 797 test subjects; ‘ABP-20m set’ includes subjects with more than ten segments collected after 20 minutes from A-line insertion; ‘NIBP-c set’ accepts only segments with an average A-line SBP/DBP and noninvasive BP (NIBP) difference of $\le 10\;\textrm{mmHg}$ in 45 seconds; and ‘ABP &NIBP set’ is an intersection of ABP-20m and NIBP-c sets.

Characteristics of dataset		Total number of subjects used in this study: 4185					
		Training set	Validation set	Test sets			
				Whole set	ABP-20m set	NIBP-c set	ABP &NIBP set
Number of surgical cases		3014	410	797	629	104	86
Number of subjects		2978	410	797	629	104	86
Number of segments		229, 323	31, 152	60, 060	29, 338	7748	3712
SBP	mean±SD$\;\textrm{mmHg}$	$111.84\pm 17.68$	$111.55\pm 17.31$	$112.07\pm 17.18$	$110.94\pm 16.26$	$108.67\pm 14.74$	$108.22\pm 14.70$
	SDS$\;\textrm{mmHg}$	19.750	19.157	19.807	15.375	19.577	15.107
DBP	mean±SD$\;\textrm{mmHg}$	$61.61\pm 11.04$	$61.76\pm 10.80$	$61.72\pm 10.92$	$60.65\pm 10.14$	$59.70\pm 9.45$	$58.45\pm 8.10$
	SDS$\;\textrm{mmHg}$	11.748	12.126	11.627	8.745	10.566	7.831
Age (mean±SD$\;\textrm{years}$)		$53.35\pm 14.86$	$53.99\pm 14.57$	$54.33\pm 14.31$	$54.67\pm 14.69$	$54.33\pm 14.72$	$54.71\pm 15.45$
Gender (Male/Female)		1410/1604	178/232	367/430	293/336	51/53	44/42

The characteristics of the cleaned subjects are summarized in Table 1. The mean and SD of the A-line SBP and DBP of 2987, 410, and 797 training, validation, and test subjects were $111.84\pm 17.68$ and $61.61\pm 11.04$, $111.55\pm 17.31$ and $61.76\pm 10.80$, $112.07\pm 17.18$ and $61.72\pm 10.92\;\textrm{mmHg}$, respectively. Three subsets of a ‘Whole’ set (the whole 797 test subjects) were constructed for a reliable test. The first subset (‘ABP-20m’) includes subjects with more than ten segments collected after 20 minutes from A-line insertion based on the rationale that ABP waveforms are probably unreliable for approximately 20 minutes following A-line insertion and ABP measurement. The second subset (‘NIBP-c’) accepts only segments with an average A-line SBP/DBP and noninvasive BP (NIBP) difference of $\le 10\;\textrm{mmHg}$ in 45 seconds (considering the cuff-measurement time) to eliminate abnormal test subjects with segments that had deteriorated by intra-measurement zeroing and transducer issues. The third subset (‘ABP &NIBP’) is an intersection of ABP-20m and NIBP-c. The ABP-20m, NIBP-c, and ABP &NIBP subsets included 629, 104, and 86 subjects, respectively (mean±SD values: $110.94\pm 16.26$, $108.67\pm 14.74$, and $108.22\pm 14.70\;\textrm{mmHg}$ for SBP and $60.65\pm 10.14$, $59.70\pm 9.45$, and $58.45\pm 8.10\;\textrm{mmHg}$ for DBP, respectively). The age distributions of training, validation, Whole, ABP-20m, NIBP-c, and ABP &NIBP sets are $53.35\pm 14.86$, $53.99\pm 14.57$, $54.33\pm 14.31$, $54.67\pm 14.74$, $54.33\pm 14.72$, and $54.71\pm 15.45$ years, respectively.

A novel metric, an SD of subject-calibration centring (SDS), was defined and measured to capture the intrasubject ABP variation level. The SDS is the SD of the ABP after the person-mean centring procedure^33^, where an initial ‘calibration’ value for each subject is used instead of the ‘mean’ value and can capture the intra-individual deviation. Therefore, this new metric can be interpreted as a design difficulty level of a calibration-based cuffless BP estimation model. The SDSs of the training SBP and DBP used in the experiment are 19.750 and $11.748\;\textrm{mmHg}$, respectively, indicating high intrasubject BP variation from an initial BP calibration. From the high SDS values of the validation SBP and DBP (19.157 and $12.126\;\textrm{mmHg}$, respectively), there is clearly high intrasubject BP variability from an initial BP calibration. In the test set, A-line SBP and DBP waveforms showed high intrasubject variation as verified by the high SDS values (19.807 and $11.627\;\textrm{mmHg}$ for SBP and DBP, respectively). The SDS values of the ABP-20m, NIBP-c, and ABP &NIBP subsets were 15.375, 19.577, and $15.107\;\textrm{mmHg}$ for SBP and 8.745, 10.667, and $7.831\;\textrm{mmHg}$ for DBP, respectively. Thus, the SDS values in our results are large enough to validate the accuracy of PPG2BP-Net with high intrasubject BP variability.

**Table 2:** BP estimation accuracy of the proposed PPG2BP-Net. Estimation was based on i) AAMI standard: The number of test subjects needs to be $\ge 85$, the ME should be $\le \pm 5\;\textrm{mmHg}$, and the SD of error should be $\le 8\;\textrm{mmHg}$; and ii) BHS standard: The grades based on the BHS standard are given based on the error percentage as follows: if $60\%$, $50\%$, and $40\%$ of errors $\le 5\;\textrm{mmHg}$, then grades A, B, and C are given, respectively; if $85\%$, $75\%$, and $65\%$ of errors $\le 10\;\textrm{mmHg}$, then grades A, B, and C, are given, respectively; if $95\%$, $90\%$, and $85\%$ of errors $\le 15\;\textrm{mmHg}$, then grades A, B, and C, are given, respectively.

Error	AAMI and BHS standards	Whole set (797 subjects)	ABP-20m set (629 subjects)	NIBP-c set (104 subjects)	ABP &NIBP set (86 subjects)
SBP error	ME	$-0.231$	0.209	$-0.415$	0.977
	SD of error	10.263	7.509	9.807	6.969
	MAE	7.991	5.525	7.752	5.238
	$\le 5\;\textrm{mmHg}$	$39.2\%$ [D]	$57.8\%$ [B]	$39.5\%$ [D]	$60.0\%$ [A]
	$\le 10\;\textrm{mmHg}$	$69.4\%$ [C]	$84.2\%$ [B]	$70.5\%$ [C]	$85.9\%$ [A]
	$\le 15\;\textrm{mmHg}$	$86.4\%$ [C]	$94.6\%$ [B]	$88.2\%$ [C]	$95.6\%$ [A]
DBP error	ME	0.062	0.150	0.699	0.519
	SD of error	6.252	4.549	5.662	4.379
	MAE	4.789	3.282	4.361	3.183
	$\le 5\;\textrm{mmHg}$	$61.7\%$ [A]	$78.7\%$ [A]	$65.8\%$ [A]	$78.0\%$ [A]
	$\le 10\;\textrm{mmHg}$	$89.3\%$ [A]	$95.4\%$ [A]	$91.3\%$ [A]	$96.6\%$ [A]
	$\le 15\;\textrm{mmHg}$	$97.6\%$ [A]	$99.1\%$ [A]	$98.3\%$ [A]	$99.2\%$ [A]

### Performance of the proposed PPG2BP-Net-based cuffless BP estimation

The PPG2BP-Net modelled by a subject-independent method was trained with 2987 subjects. A sufficient number of training and test subjects can circumvent misleading results in the holdout validation. After training completion, the estimation accuracies of the ME, SD of error, and mean absolute error (MAE) off 797 Whole, 629 ABP-20m, 104 NIBP-c, and 86 ABP &NIBP test subjects were evaluated (Table 2). Compared to the AAMI standard, the test subsets fulfil all requirements (i.e., the test sample size needs to be $\ge 85$; ME should be $\le \pm 5\;\textrm{mmHg}$; and SD of error should be $\le 8\;\textrm{mmHg}$), except for Whole and NIBP-c sets whose SDs of estimated SBP error are 10.263 and $9.807\;\textrm{mmHg}$, respectively. The results with test subjects acquired after 20 minutes from A-line insertion and ABP monitoring (ABP-20m and ABP &NIBP sets) revealed that PPG2BP-Net performance thoroughly met the AAMI standard. The ME and SD of error (ME±SD) between the ground-truth A-line SBP and its estimated values obtained via the proposed PPG2BP-Net with ABP-20m and ABP &NIBP test subjects are $0.209\pm 7.509$ and $0.977\pm 6.969\;\textrm{mmHg}$, respectively, and the estimated DBP are $0.150\pm 4.549$ and $0.519\pm 4.379\;\textrm{mmHg}$, respectively. Of note, the PPG2BP-Net tested with an ABP &NIBP set achieved Grade A BHS standard for all categories with complete agreement with the AAMI standard. The grades based on the BHS standard based on the error percentage are as follows: if $60\%$, $50\%$, and $40\%$ of errors $\le 5\;\textrm{mmHg}$, then grades A, B, and C are given, respectively; if $85\%$, $75\%$, and $65\%$ of errors $\le 10\;\textrm{mmHg}$, then grades A, B, and C, are given, respectively; and if $95\%$, $90\%$, and $85\%$ of errors $\le 15\;\textrm{mmHg}$, then grades A, B, and C, are given, respectively. Noting that the SDS values of the Whole and NIBP-c sets are excessively larger than others (Table 1), we discern the rationale of SBP’s higher SD of error than that of DBP and that SBP estimation is more challenging than DBP estimation, which was further verified from the BHS standard, where grade A is obtained for whole DBP error distribution, but only for parts of the SBP error categories. From tables 1 and 2, we surmise that the proposed PPG2BP-Net would be a guideline for designing the C3A cuffless BP-estimation devices for accurate BP estimation from newly measured actual PPG data.

### Comparative study

**Table 3:** Performance comparison among various learning- and PPG-based cuffless BP estimation systems based on the AAMI standard. The highlights in bold represent a subject-dependent modeling and the violation of the AAMI standard.

Learning algorithms for BP estimation	Modeling & experiment	Data Source	Number of training subjects		Number of validation / test subjects		SBP error ($\textrm{mmHg}$)		DBP error ($\textrm{mmHg}$)	
					Subject exclusive from training subjects		ME	SD of error	ME	SD of error
13’DBN-RBM^17^	Subject-independent	In-house	525		${\textbf {0}}$ / ${\textbf {47}}$ **(holdout)**		$-2.98$	${\textbf {19.35}}$	$-3.65$	${\textbf {8.69}}$
16’ANN^18^	**Subject-dependent**	MIMIC II	$70\%$ of 69		${\textbf {15}}\%$ / ${\textbf {15}} \%$ **of** ${\textbf {69}}$ **(holdout)**		0.06	7.08	0.01	4.66
16’SVR^19^	**Subject-dependent**	In-house	32		10-fold validation		4.77	7.68	3.67	5.69
16’SVR^20^	**Subject-dependent**	In-house	65		10-fold validation		${\textbf {5.1}}$	4.3	4.6	4.3
19’DTR^24^	**Subject-dependent**	MIMIC II	at least 441		10-fold validation		0.021	${\textbf {18.543}}$	$-0.247$	6.736
19’SVR^24^							$-0.903$	${\textbf {16.717}}$	$-0.655$	7.506
19’RFR^24^							0.155	${\textbf {10.683}}$	0.196	4.731
19’AdaboostR^24^							$-0.050$	${\textbf {8.901}}$	0.187	4.173
19’RFR^26^	Subject-independent	In-house	$<50\;\textrm{yr}$	418	SBP$<120\;\textrm{mmHg}$	leave one out validation	${\textbf {6.3}}$	7.2	3.6	6.7
				257	$120\le \text {SBP}\le 139\;\textrm{mmHg}$		$-3.9$	7.2	$-2.4$	7.3
				${\textbf {64}}$	SBP$\ge 140\;\textrm{mmHg}$		${\textbf {-20.2}}$	${\textbf {14.2}}$	${\textbf {-10}}$	${\textbf {11.7}}$
			$\ge 50\;\textrm{yr}$	364	SBP$<120\;\textrm{mmHg}$		${\textbf {12.8}}$	${\textbf {9}}$	4.2	7.0
				574	$120\le \text {SBP}\le 139\;\textrm{mmHg}$		0.5	${\textbf {8.2}}$	0.5	7.8
				402	SBP$\ge 140\;\textrm{mmHg}$		${\textbf {-14.6}}$	${\textbf {11.5}}$	$-2.9$	${\textbf {8.9}}$
20’CNN-LSTM^28^	**Subject-dependent**	MIMIC II	140		${\textbf {20}}$ / ${\textbf {40}}$ **(holdout)**		1.91	5.55	0.67	2.84
20’LRCN^29^	**Subject-dependent**	UCI DB	1557		10-fold validation		1.55	5.41	$-1.25$	5.65
21’CNN^27^	Subject-independent	UCI DB	1620		10-fold validation		1.64	7.42	$-0.28$	5.81
22’RFPASN^30^	**Subject-dependent**	MIMIC II	1562		10-fold validation		0.0086	3.2865	$-0.0492$	2.4002
22’Concat-CNN^31^	**Subject-dependent**	MIMIC-II	140		${\textbf {20}}$ / ${\textbf {40}}$ **(holdout)**		$-0.15$	5.26	$-0.29$	2.60
CNN-based PPG2BP-Net (Proposed)	Subject-independent	In-house (operation room)	2987		410 / 797 (whole: holdout)		$-0.231$	${\textbf {10.263}}$	0.062	6.252
					410 / 629 (ABP-20m: holdout)		0.209	7.509	0.150	4.549
					410 / 104 (NIBP-c: holdout)		$-0.415$	${\textbf {9.807}}$	0.699	5.662
					410 / 86 (ABP &NIBP: holdout)		0.977	6.969	0.519	4.379

**Table 4:** Characteristics of the subject data in the UCI DB from the MIMIC II dataset (i.e., dataset used in 21’CNN^27^) after the preprocessing shown in Fig. 1.

Characteristics of dataset		UCI DB from MIMIC II
Number of surgical cases		2958
Number of subjects		2958
Number of segments		77,418
SBP	mean ± SD$\;\textrm{mmHg}$	$128.61\pm 20.3$
	SDS$\;\textrm{mmHg}$	7.509
DBP	mean ± SD$\;\textrm{mmHg}$	$66.16\pm 10.1$
	SDS$\;\textrm{mmHg}$	4.127

In Table 3, the various PPG-based BP estimation systems were compared to the proposed PPG2BP-Net. The table includes the learning models, experimental methods, data source, number of subjects in training and validation(test) sets, and the BP-estimation accuracy. As shown in Table 3, earlier studies^17,24,26^ did not fulfil the AAMI requirements. Some studies^18,19^ in 2016 met AAMI standard, yet similar performance was doubtable for exclusively acquired actual PPG data of a new subject because the number of subjects involved in the training and validation was the minimal number ($<100$) of subjects. Recent studies^29,30^, between 2021 and 2022, used approximately 1600 subjects, but do not guarantee satisfactory performance with the exclusively measured actual PPG data as the learning systems were modelled and validated through a subject-dependent method. Other recent BP estimation systems^28,31^ were verified by the holdout validation and fulfilled the AAMI requirements, yet accurate BP estimation from exclusively measured actual PPG data would not be guaranteed owing to the lack of subjects used in the training and validation (i.e., 140 and 20 subjects, respectively). A CNN-based scheme^27^ with 1620 subjects from Multiparameter Intelligent Monitoring in Intensive Care (MIMIC) II dataset with subject-independent modelling and experiment is comparable to our scheme, though their estimation performance was slightly worse than ours despite a smaller sample. Furthermore, the intrasubject ABP deviation is relatively low (Table 4). The SDS values of University of California, Irvine (UCI) database (DB) from MIMIC II dataset are analysed after the same data preprocessing as that in this study. When compared to the SDS values of the dataset used in this study ($19.750\;\textrm{mmHg}$ and $11.748\;\textrm{mmHg}$ for SBP and DBP, respectively; Table 1), the SDS values of UCI DB from the MIMIC II dataset are considerably low (i.e., 7.509 and $4.127\;\textrm{mmHg}$). The low SDS values represent the low variation of BP within a subject, which may cause a *nonregenerative issue* for estimating highly varying intrasubject BP, and because the SD of error becomes identical to the SDS value if the estimated BPs are intentionally/accidently set to the calibration BP without actual estimation, then the AAMI standard (SD of error $\le 8\;\textrm{mmHg}$) is always fulfilled if the SDS $\le 8\;\textrm{mmHg}$, i.e., an *overqualified issue*.

## Discussion

To precisely design a learning-based BP estimation model such that its estimation accuracy obtained during the test is sustained after being built upon a practical cuffless BP monitoring system (i.e., for the model-generation capability), the following delicate yet realistic experimental principles are applicable: i) the number of subjects should be sufficiently large, ii) subject independent training and test datasets are required, and iii) the intrasubject BP variation should be carefully scrutinized in the model design.

First, for a new subject, the PPG-based BP estimation accuracy can be improved as the number of subjects used in the modelling increases, because the model can learn PPG features that dynamically change according to the BP variation. In many previous studies on learning-based cuffless BP estimation, the PPG waveforms were acquired from the MIMIC II database^18,21,24,27–31^. Recently, the training subject number has increased to $\ge 1000$ (e.g., 1557, 1562, and 1620 in the training of LRCN^29^, RFPASN^30^, and CNN^27^, respectively). Conversely, the datasets can be acquired for specific studies of the learning systems (e.g., the Critical Care Department and the Post-Anesthesia Care Unit of Vall d’Hebron University Hospital in Barcelona, Spain^17^, the University of Queensland Vital Signs Dataset^19^, the Tsinghua University^20^, Royal Adelaide Hospital^22,23^, and Suzhou Hospital of Nanjing Medical University^26^). However, the number of subjects in the in-house dataset was generally smaller (i.e., $<1000$) than that acquired from MIMIC II. In this study, we used 2987 subjects, cleaned from the raw, vital waveforms of 25, 779 surgical cases acquired by SNUH^32^.

Second, if the PPG samples from an identical subject are used for both the training and test datasets, the model would be overfitted to the subject, and to prevent overfitting in the model generation, a subject-independent dataset is needed (i.e., the training and test datasets should be structured from different subjects). Further, a widely used non-exhaustive cross-validation strategy, i.e., a ‘holdout’ method, was employed for the validation and test, and this strategy randomly divides the original data into the training and test sets (a.k.a., a holdback set): commonly $80\%$ and $20\%$, respectively. Contrary to a *k*-fold validation strategy that tests multiple times and averages the test results, the holdout method involves a single validation (test), which may mislead the evaluation result. Thus, the holdout strategy is relevant only when the samples in the training dataset is sufficient to avoid misleading results. Conversely, if the number of PPG datasets is sufficient, then the learning-based BP estimation systems tested by the fully independent validation data can accurately estimate BP from the exclusively measured and never-seen actual PPG data with a higher probability. Accordingly, the fidelity of the BP estimation with currently measured PPG can be improved by a learning-based BP estimation system certified through a ‘holdout’ method with the ‘sufficient number of subjects’.

Third, for a calibration-based BP estimation model, accuracy performance could be overqualified if the intrasubject BP variation is low. Moreover, a learning model could be nonregenerative if it is strongly biased to the BP calibration even when trained and validated with highly varying inter-subject BP (i.e., subject-dependent). Thus, a reliable calibration-based cuffless BP estimation is not necessarily guaranteed for a new subject with highly varying BP. To circumvent the overqualified and nonregenerative issues, the proposed novel metric (i.e., SDS) can be used to validate the accuracy of a subject’s calibration-based highly varying BP estimation. Note that the typical SD metric can characterize cardiovascular dynamics among subjects, yet cannot capture the intra-individual deviation to validate the calibration-based cuffless BP estimation. In addition, there exists ambiguity on the subject-wise SD metric to validate the calibration-based BP estimation model. However, the proposed novel SDS values metaphorically represent the inter-subject ABP deviation from the initial calibration BP. Therefore, the high value of an SDS metric implies that the ABP estimation is more challenging because the initially calibrated ABP is used for the estimation of the highly varying target BP which has high discrepancy to the initial calibration value. Furthermore, the high deviation of ABP within a subject is implicitly dissolved into the SDS metric. In the final analysis, the SD metric is relevant merely for the calibration-free BP estimator’s performance evaluation, and not for the calibration-based BP estimator. In contrast, the proposed SDS metric can be used to quantify the performance of a subject’s calibration-based highly variable BP estimation.

## Methods

### Approval for data collection using vital recorder

The data collection of the VitalDB^34^ dataset has been approved by the institutional review board (IRB) of SNUH (IRB no. 1408-101-605), and the construction of the data repository was registered at a publicly accessible clinical trial registration site (ClinicalTrial.gov, NCT02914444). The retrospective analysis of the registry was approved by the SNUH IRB (no. 2004-120-1118). We confirm that this research has been performed in accordance with the following three guidelines: i) STROBE(STrengthening the Reporting of OBservational studies in Epidemiology) guidelines; ii) Guidelines for developing and reporting machine learning predictive models in biomedical research: a multidisciplinary view; and iii) Declaration of Helsinki ethical principles for medical research involving human subjects. This study was exempted by IRB of SNUH (IRB no. 1408-101-605) from the requirement of informed consent from the patient due to the retrospective study design.

The vital waveforms to build a BP estimation system in this study include not only the ABP and PPG waveforms measured by TramRac-4A (GE Healthcare) but also the ABP- and NIBP-SBP/DBP waveforms measured by Solar 8000M (GE Healthcare). Anaesthesia-related information was collected by Primus (Dr$\ddot{a}$ger) and Orchestra (Fresenius Kabi) to extract the ABP, NIBP, and PPG data after anaesthetization and before the surgery. A Vital Recorder^32^ aggregated the measured raw data as either a waveform with a sampling frequency of $500\;{\textrm{Hz}}$ or a numeric. Moreover, demographic information, such as age, height, and weight of the subjects in the surgical cases, was recorded to check the fidelity of the acquired data.

### SDS metric calculation

**Figure 2:**
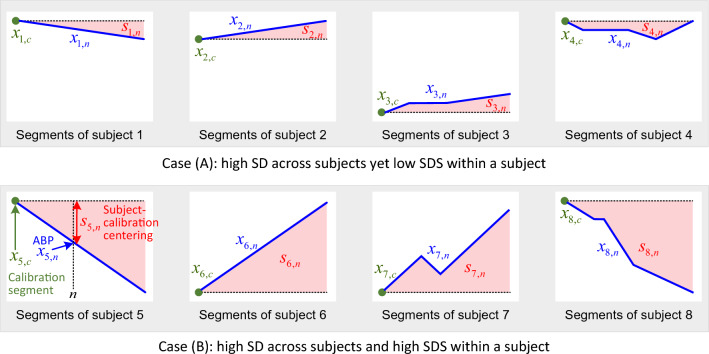
The SD and SDS of BP dynamics. Case (**A**) represents high BP deviation across the subjects, but with low intrasubject deviation. Case (**B**) represents high BP deviation both across and within subjects. SD does not distinguish between cases (**A**) and (**B**), whereas SDS can clearly distinguish these cases.

There is ambiguity on the subject-wise SD metric to validate the calibration-based BP estimation model, as we can see by comparing the extreme cases as illustrated in Fig. 2: Case (A) represents high BP deviation across the subjects yet low deviation within a subject. Case (B) represents high BP deviation across the subjects as well as within a subject. To eliminate the ambiguity on the subject-wise SD by quantitatively measuring the intrasubject BP variation, a subject-calibration centring ABP was defined as follows:

1 $$\begin{aligned} s_{i,n}=x_{i,n}-x_{i,c}, \end{aligned}$$

where $x_{i,n}$ denotes the *n*th segment ABP of subject *i* and $x_{i,c}$ is the ABP used for the calibration of subject *i*. Comparing to a conventional SD metric, the SDS metric of ABP is then defined as follows:

2 $$\begin{aligned} {\text {SD}}= & {} \sqrt{\frac{1}{\sum _i N_i -1} \sum _{i}\sum _{n=1}^{N_i} \left( x_{i,n}-{\bar{x}}\right) ^2 }, \end{aligned}$$

3 $$\begin{aligned} {\text {SDS}}= & {} \sqrt{\frac{1}{\sum _i N_i-1} \sum _{i}\sum _{n=1}^{N_i} \left( s_{i,n}-{\bar{s}}\right) ^2}, \end{aligned}$$

where $N_i$ is the number of segments of subject *i*. Here, ${\bar{x}}$ and ${\bar{s}}$ are the mean values of $x_{i,n}$ and $s_{i,n}$, respectively, for all subject *i*’s and segment *n*’s, which are obtained as follows:

4 $$\begin{aligned} {\bar{x}}= & {} \frac{1}{\sum _i N_i} \sum _{i}\sum _{n=1}^{N_i} x_{i,n}, \end{aligned}$$

5 $$\begin{aligned} {\bar{s}}= & {} \frac{1}{\sum _i N_i} \sum _{i}\sum _{n=1}^{N_i} s_{i,n}. \end{aligned}$$

### Data preprocessing

**Figure 3:**
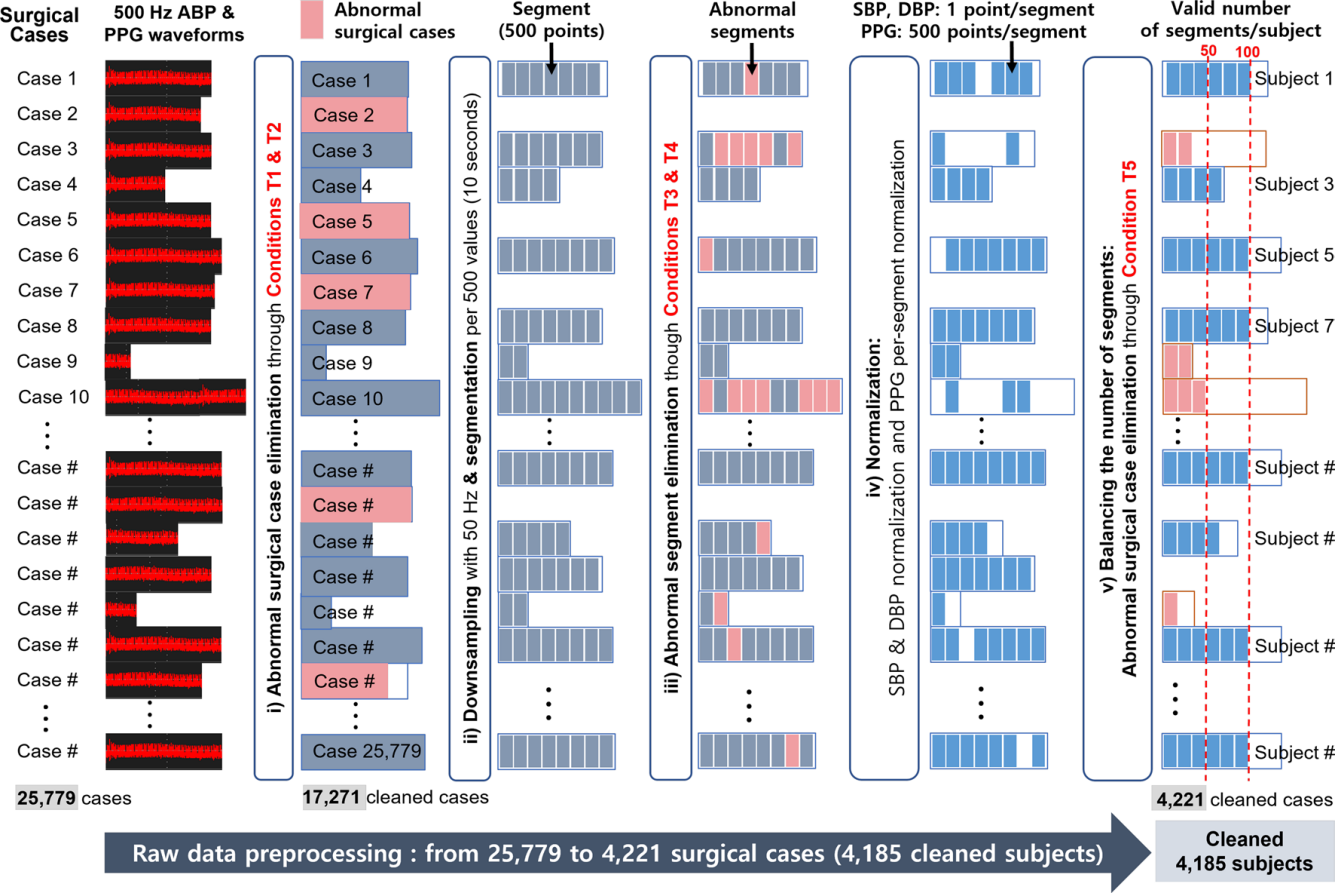
Data preparation for PPG2BP-Net training and validation. From the ABP and PPG raw data of 25, 779 surgical cases, cleaned and independent data for 4185 subjects of 4221 surgical cases were obtained.

The details of data preprocessing are depicted in Fig. 3.

#### Abnormal surgical case elimination

During the data acquisition, the additive thermal noise was precancelled through a filter in the data acquisition devices introduced in ‘Approval for data collection using vital recorder’ subsection. However, abnormal and redundant data could be blended into the raw data (e.g., outlier data from subjects in exceptional conditions and almost identical ABP and PPG data). As the unreliable raw data hindered our PPG2BP-Net from learning effectively, the raw data cleaning-and-preprocessing procedure is essentially required to build an effective and efficient learning-based BP estimation system. Further, to reject the abnormal cases from the raw ABP and PPG datasets, as the first step, Conditions T1 and T2 for the clean (reliable) cases are considered as follows:

- T1: The condition of cases should be unexceptional such that $10\le {\text {weight}}\le 100\;\textrm{kg}$, $100\le {\text {height}}\le 200\;\textrm{cm}$, $18\le {\text {age}}\le 100\;\textrm{years}$, and nonpregnant;
- T2: Essential information (e.g., operation time log, PPG, and ABP) should be included.

If any of the criteria in Conditions T1 and T2 is violated, then the corresponding case is eliminated because the exceptional cases, e.g., weight$<10~\;\textrm{kg}$ or height$<100~\;\textrm{cm}$, are sparse and unreliable, resulting in inefficient training. Throughout this step, 469 and 8040 abnormal surgical cases were eliminated based on T1 and T2, respectively, and 17, 271 clean cases were obtained. Here, the T2 violation was mainly caused by null data with no wearing a PPG or ABP device.

#### Downsampling and segmentation

As the size of the data sequence increases, the training complexity also increases. Moreover, since the training performance depends on the training data size, an appropriate size of data should be designed. Thus, after the elimination of the abnormal cases, the $500\;\textrm{Hz}$-sampled ABP and PPG data of the remaining cleaned subjects are downsampled and segmented to train the designed PPG2BP-Net efficiently. Concretely, the $500\;\textrm{Hz}$-sampled data are downsampled with $50\;\textrm{Hz}$ and then segmented into multiple segments, each comprising 500 points (i.e., the 10-second data per segment). Consider the following example: an 8-seconds length segment was used to design ANN^18^ and LRCN,^29^ and a 10-seconds length segment, referred to as a frame, was used to design SVR.^19^ The non-overlapped segmentation was performed to maximize the information in the collected data.

#### Abnormal segment elimination

In this step, the abnormal PPG and ABP segments (caused by movement artifact, not wearing a PPG or ABP device, and so forth) and sparse case segments (e.g., SBP$>180\;\textrm{mmHg}$) are eliminated because they decrease BP estimation accuracy and make training inefficient. Segments with invalid pulse rate, abnormal SBP/DBP fluctuation or irregular pulse are excluded. The additional clean segment conditions (i.e., Conditions T3 and T4) are as follows:

- T3: PPG & ABP segments should include only valid data: no null value and at least one non-zero data;
- T4: ABP segments of typical SBP: $70\le \text {average SBP}\le 180\;\textrm{mmHg}$.

If any of the criteria in Conditions T3 and T4 is violated, then the corresponding segment is eliminated from the subject. Similarly, the abnormal segments are eliminated from all subjects.

#### Normalization

The A-line SBP and DBP comprise the average values of the systolic peak pressure and end-diastolic pressure in each A-line pulse. The SBP and DBP values are standardized with the mean and SD of the entire training set. This normalization step can improve the learning accuracy.

#### Balancing the number of segments

Normalized subjects with smaller than 50 clean segments, of which is 13, 050 surgical cases, are discarded based on Condition T5:

- T5: The numbers of remaining clean PPG and ABP segments after an ‘abnormal segment elimination’ step should be greater than or equal to 50.

If a subject has more than 100 clean segments, then randomly selected 100 clean segments are retained in the subject. Thus, each every remaining subjects include a balanced number of normal PPG and ABP segments between 50 and 100, so that they can fairly affect the training and validation.

### Proposed PPG2BP-net

#### Subject-wise batch construction on train

Considering the computational complexity of the learning and the capability of a central processing unit in a simulation computer, a training batch is constructed with 64 segments (rather than 128 and 256) from the clean training sets. To learn in various cases, the 64 independent subjects were randomly and repetitively selected from 2987 training subjects to train the proposed PPG2BP-Net in various cases. The training procedure is summarized in Algorithm 1.
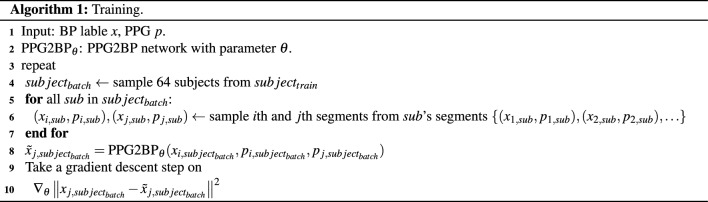

**Figure 4:**
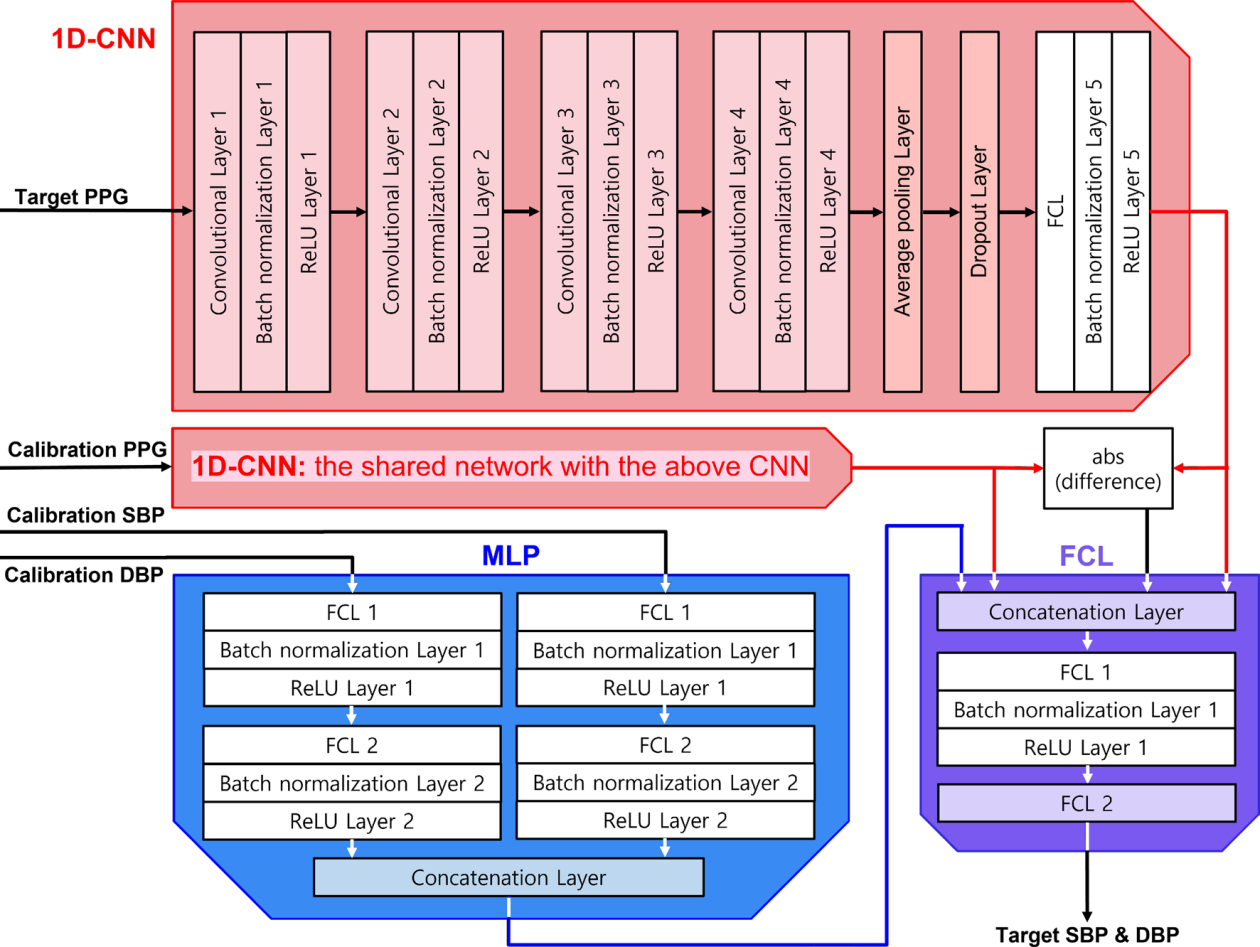
Proposed PPG2BP-Net that comprises a comparative paired one-dimensional CNNs, one MLP, and one FCL.

One segment is randomly selected from each selected subject for the target segment, $(x_{j,sub},p_{j,sub})$, and another segment, $(x_{i,sub},p_{i,sub})$, is selected for the calibration information. The random subject and segment selections are used for moderately training PPG2BP-Net with identical weights across the training subjects, which is a similar purpose to that of balancing the number segments. The hyperparameters, namely, the learning rate and the number of epochs, are stochastically determined during the learning based on the initial learning rate 0.0001 and within the maximum number of epochs 1000.

The detailed structure of the proposed PPG2BP-Net is depicted in Fig. 4.

#### 1D-CNN architecture

The proposed learning system utilizes 1D-CNNs of shared network with the same structure and parameters as the main feature extraction networks (Fig. 1). The clean $1\times 500$ calibration PPG segment vectors are fed into an 1D-CNN as the input for training, and the target PPG sequences go through the other paired 1D-CNN as input. From the designed paired structure of two 1D-CNNs, the network can effectively learn the varying relationship between the target and calibration PPGs. Further, the difference between the two features of the two 1D-CNNs is also learned in a fully connected layer (FCL). Thus, the designed 1D-CNN structure is called a comparative paired 1D-CNN structure.

Concretely, the proposed 1D-CNN model includes four hidden layer groups followed by an average pooling layer and a dropout layer. Each hidden group comprises one convolutional layer, a batch normalization layer, and a rectified linear unit (ReLU) layer. The hidden non-linear features can be implicitly extracted by four alternating convolutional and ReLU layers. To capture the time series of features in the PPG waveforms, 1D-CNN is employed, and multiple filters are employed because a single one-dimensional filter is insufficient to effectively extract the unknown and various features from the calibration PPG waveforms. The batch normalization between the convolutional and ReLU layers normalizes the hidden layer input and resolves an issue caused by change in the input distribution^35^. At the end of each hidden layer, the most widely used activation function (i.e., a ReLU) is employed^36^ for better and faster learning.

The waveforms after the fourth hidden layer group are sampled through an average pooling layer, which can reduce the network’s complexity by sustaining the essential information of the features. The $30\%$ output data in the average pooling layer are dropped out (set to zero) in the dropout layer by randomly removing $30\%$ of neurons during the training (i.e., hyperparameter dropout rate: 0.3). The dropout prevents a nonsensical action from significantly relying on a particular input and thus reduces over-fitting and enhances generalization^37^. After the dropout layer, each batch goes through an FCL with the eight units and is normalized in a batch normalization layer such that the mean and variance are zero and one, respectively, to improve the convergence speed and learning performance^35,38^.

Two 1D-CNN output sequences and their absolute difference will be provided to the final FCL module (Fig. 4), as input, and would then be activated by a ReLU function.

#### Multilayer perceptron

A multilayer perceptron (MLP) is employed to assist feature extraction for the supervised learning from the numeric feature data, namely, the A-line SBP and DBP values. As shown in the left-hand bottom side of Fig. 4, the calibration SBP and DBP values are separately provided into two FCLs, and their features are extracted. Each FCL is followed by a batch normalization layer and a ReLU layer. The two output features from the independent ReLU layers are gathered and concatenated. The concatenated features are fed in a final FCL module as one of the four inputs to estimate the target SBP and DBP (right-bottom panel, Fig. 4).

#### FCL

The adaptive feature learning is completed at a final FCL (right-bottom panel, Fig. 4). The output features from two 1D-CNNs, the difference between them, and MLP are concatenated. The single output sequence of the concatenation layer is then provided to an FCL, followed by a batch normalization layer and a ReLU layer. The output of the ReLU layer produces the target SBP and DBP through another FCL.

#### Validation and test

The proposed PPG2BP-Net-based cuffless BP measurement system uses two sets of calibrations. In the experiment, the first and second segments of PPG, SBP, and DBP are used as the calibration segments for validating or testing the remaining independent segments. The estimated SBP and DBP of a target segment in each subject are the average values of estimated SBPs and DBPs, respectively, with the calibration PPG, SBP, and DBP in the first and second segments. The ground-truth SBP and DBP are the average values of the two calibration segments. The validation or test procedure is summarized in Algorithm 2. Here, re-calibration^10^ is not considered as for the C3 BP estimation, though it can improve the BP estimation accuracy.
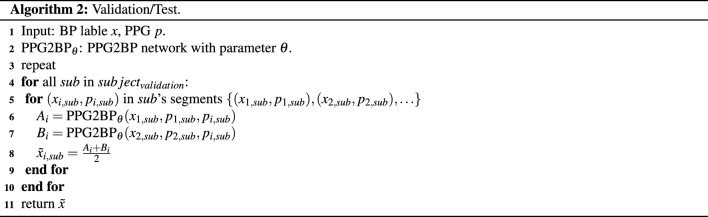


Since the predictable BP range of the proposed PPG2BP-Net is restricted between $70\;\textrm{mmHg}$ and $180\;\textrm{mmHg}$ based on T4, to enlarge the predictable BP range, an additional learning process is required with sufficient and reliable data of BP less than $70\;\textrm{mmHg}$ and greater than $180\;\textrm{mmHg}$. Further verification of the designed PPG2BP-Net through a clinical test will enhance the fidelity of the proposed C3A cuffless BP estimation. In this case, a certified cuff-based BP device can be used to obtain the calibrations.

## Data Availability

The part of the collected vital signs can be found in the database VitalDB^34^ (https://vitaldb.net/dataset/?query=api). The code used in this study is a private asset (protected by intellectual property) that was developed and is owned by Sky Labs Inc. Thus, the code used in this study will be made partially available by the corresponding author C. Choi upon reasonable request.
